# Quadriceps-sparing versus traditional medial parapatellar approaches for total knee arthroplasty: a meta-analysis

**DOI:** 10.1186/s12891-019-2482-7

**Published:** 2019-03-20

**Authors:** Fu-Zhen Yuan, Ji-Ying Zhang, Dong Jiang, Jia-Kuo Yu

**Affiliations:** Institution of Sports Medicine, Beijing Key Laboratory of Sports Injuries, Peking University Third Hospital, Beijing, 100191 China

**Keywords:** Knee arthroplasty, Meta-analysis, Minimally invasive, Quadriceps-sparing, Medial parapatellar

## Abstract

**Background:**

There is still controversy regarding whether Quadriceps-sparing (QS) approach for total knee arthroplasty (TKA) lead to better earlier recovery as well as compromising low limb alignment and prosthesis position compared with conventional medial parapatellar (MP) approach. To overcome the shortcomings and inaccuracies of single studies, the clinical outcomes and radiographic assessments of QS approach and MP approach were evaluated through meta-analysis.

**Methods:**

We performed this meta-analysis according to the Preferred Reporting Items for Systematic Reviews and Meta-analysis guidelines. A literature search was conducted in the PubMed, EMBase, Cochrane Collaboration Library and Web of Science databases. Our search strategy followed the requirements of the Cochrane Library Handbook. The study selection, data extraction and assessment of methodological quality were independently completed by four authors. And subgroup analysis and publication bias were also performed in the study.

**Results:**

Eight prospective randomized controlled trials (RCTs) and eight retrospective studies were identified. Overall meta-analysis and subgroup meta-analysis of RCTs identified the QS approach mainly was associated with increased Knee Society function score beyond 24 months postoperatively (weighted mean difference [WMD] 1.78, *P* = 0.0004) (WMD 1.86, *P* = 0.0002), and improved range of motion 1–2 weeks postoperatively (WMD 5.84, *P* < 0.00001) (WMD 4.87, *P* = 0.002). Besides, lower visual analogue scale on postoperative day 1 (WMD -0.91, *P* = 0.02), shorter hospital stay (WMD -0.88, *P* = 0.02) and shorter incision (extension) (WMD -4.62, *P* < 0.00001) were indicated in overall meta-analysis. However, surgical and tourniquet time was significantly longer in QS group by both overall and subgroup meta-analysis.

**Conclusions:**

QS approach may accelerate early recovery without increasing the risk of malalignment of low limb and malposition of prosthesis.

## Background

Total knee arthroplasty (TKA) was first performed in 1968 [1]. It is widely used in patients with symptomatic, end-stage knee arthritis [2–4] and is the most successful surgical procedure for relieving pain and improving poor function in patients with advanced arthritis [5, 6]. The conventional medial parapatellar (MP) approach has been established as the gold standard technique for TKA [7–11]. However, since the first quadriceps-sparing (QS) approach was performed in 2002 [12], it has become one of the most common alternatives to the MP approach and, theoretically, provides a faster recovery of muscle. By avoiding violation of the extensor mechanism and suprapatellar pouch and everting the patella, the QS approach aims to produce less discomfort, provide a faster recovery and reduce the extent of patellar devascularization that can lead to patellar subluxation, dislocation, avascular necrosis, fracture, patellar component loosening, and anterior knee pain [13]. Currently, numerous well-designed studies have compared the outcomes of the QS and MP approaches. However, the conclusions from studies are still controversial. Some studies have found no significant differences between the two approaches [14, 15], whereas others have supported either the QS [16–23] or the MP approach [24–26]. Therefore, we designed this meta-analysis to quantitatively compare the efficacy and safety of the QS versus the MP approach for TKA.

## Methods

Our meta-analysis was conducted according to the Preferred Reporting Items for Systematic Reviews and Meta-analysis (PRISMA) statement that established procedures for rigorous performance and reporting of meta-analyses [27, 28].

### Search strategy

Two authors independently carried out a systematic search (last update 4 August 2018) of the PubMed, EMBase, Cochrane Collaboration Library and Web of Science databases, without restrictions on regions, publication types, or languages. The following search strategies were used in the search: #1. (knee arthroplasty) OR knee replacement; #2. (((((quadriceps-sparing) OR quadriceps sparing) OR quad-sparing) OR quad sparing) OR minimally invasive) OR mini-incision; #3. #1 AND #2. Furthermore, the references from all accessed papers were also searched for any undetected studies. The results of our database search were imported into EndNote X7 and duplicates were eliminated using the duplicate removal function. Then, two authors screened all entries by title and abstract, and the remaining studies underwent full text review.

### Inclusion and exclusion criteria

Studies were selected on the basis of the following criteria: (1) study design: randomized controlled trials (RCTs), and retrospective comparative studies (both cohort and case-control studies); (2) study population: adult patients who underwent primary TKA; (3) intervention: including both QS TKA and MP TKA; (4) available mean and standard deviation (SD) or proportion (or ability to estimate SD using data range). Review articles, case reports, editorials, letters to the editor, animal experimental studies and cadaver studies were excluded.

### Data extraction and methodological quality assessment

Data were extracted using a predesigned sheet that included authors, publication data, specific interventions, main participant characteristics and results by three authors. Unreported data needed for this meta-analysis were obtained by communicating with the author though e-mail. For methodological quality evaluation of RCTs, recommendations issued by the Cochrane Handbook for Systematic Reviews were utilized in the meta-analysis [29]. The methodological quality of the included nonRCTs were evaluated with the modified Newcastle-Ottawa Scale (NOS), a simple tool used for the assessment of case controlled and cohort studies [30] that has been recommended by Cochrane collaboration [29]. NOS consists of three factors: patient selection, comparability of the study group and assessment of outcomes. According to NOS, a study can be awarded 0–9 stars.

### Statistical analysis

This meta-analysis was performed with Review Manager 5.3 (Cochrane Collaboration, Oxford, UK). The level of significance was set at *P* < 0.05. For dichotomous outcomes, the odds ratio (OR) and 95% confidence interval (95% CI) were calculated. For continuous outcomes, weighted mean difference (WMD) and the 95% CI were calculated. Statistical heterogeneity was tested with the *I*^*2*^ statistic and the Chi-squared(*χ*^*2*^)test. A *P* > 0.1 and an *I*^*2*^ ≤ 50% were considered to represent the absence of statistical heterogeneity. If significant heterogeneity (*I*^*2*^ > 50%) was found in the meta-analysis, a random effects model was used, otherwise, a fixed effects model was employed [29]. Certain studies in this meta-analysis provided data ranges (maximum and minimum values) rather than SDs. In these instances, SD was estimated as the difference between the maximum and minimum values divided by four [31], which serves as a conservative estimate of SD. Sensitivity analyses were conducted on the different types of study designs and the different participants enrolled in studies. Funnel plots were used to screen for potential publication bias.

## Results

### Literature search

**Fig. 1:**
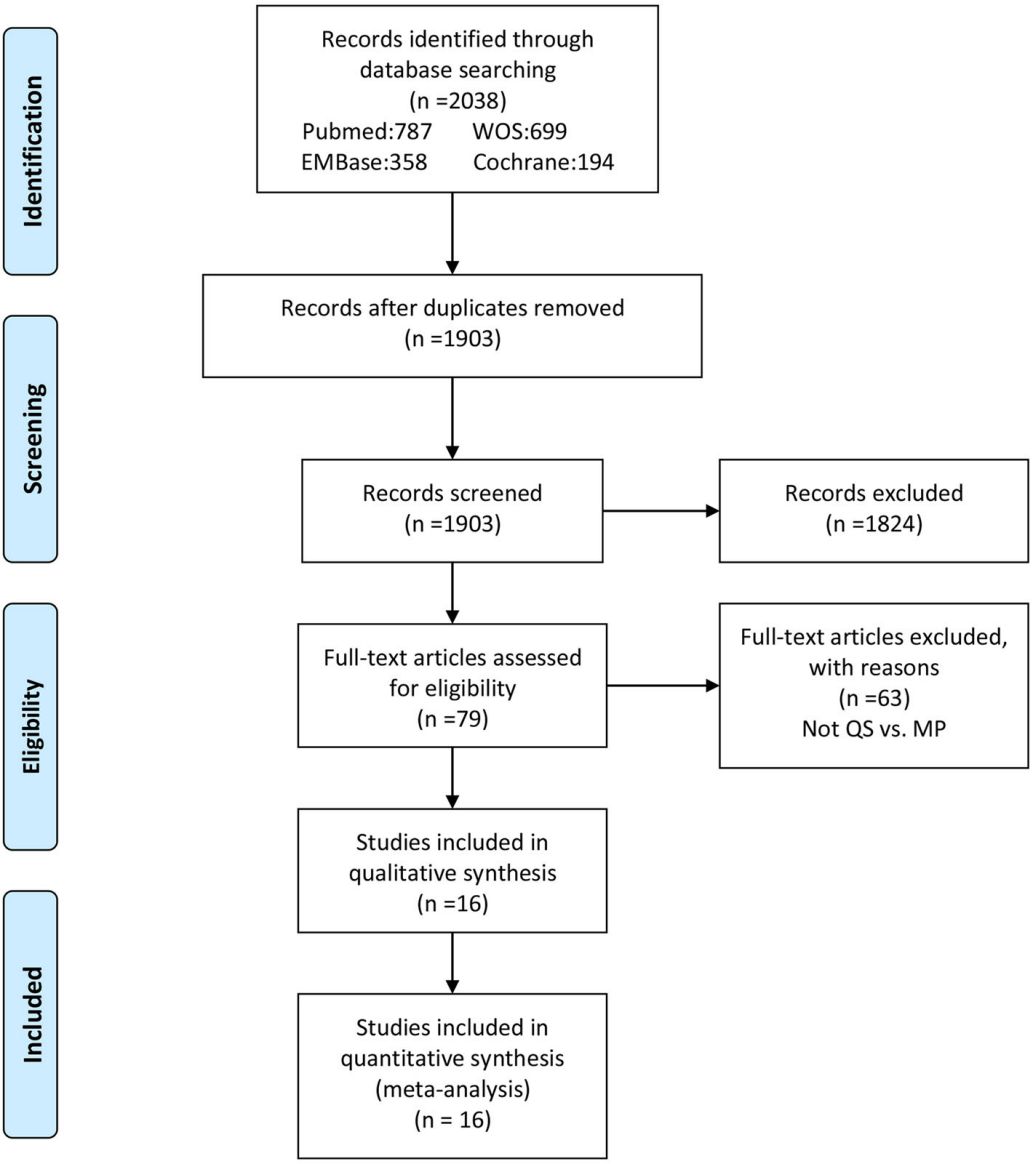
PRISMA flow chart of literature screening

The details of identifying relevant studies are shown in a flow chart of the study selection process (Fig. 1). The initial search identified 2038 potentially relevant citations from PubMed, EMBase, Cochrane Collaboration Library and Web of Science. After the duplicates were removed, 1903 studies were identified. A total of 1824 records were excluded based on a review of abstracts, leaving 79 articles for full-text review. Following full-text review, 16 citations were finally included consisting of eight RCTs [15, 16, 21, 24, 25, 32–34] and eight non-RCTs [14, 17–20, 23, 26, 35].

### The characteristics of included studies

Table 1 summarizes the key characteristics of the included studies. There was a total of 1112 patients with 1439 TKAs in the included studies. The mean age of the included patients ranged from 42 to 88 years, the mean BMI ranged from 17.9 to 49 kg/m^2^, and the mean follow-up duration ranged from 0 days to more than five years. Seven studies favored the QS approach results, while nine studies favored the MP approach results.

**Table 1:** Characteristics of included studies

Study/Year	Country	Recruitment period	Group	Patients (male/female)	Number of TKAs	Age (year)	BMI (kg/m^2^)	Follow-up (months)	Results Favor
Huang 2016	China	2005–2007	QS	2/29	31	69.3 ± 7.9	26.9 ± 3.3	65 ± 3.8	QS
			MP	2/28	30	71.2 ± 5.8	26.7 ± 2.8	68 ± 5.4	
Qi 2016	China	2005–2007	QS	2/26	30	65.3 ± 6.9	26.5 ± 3.0	74.8	QS
			MP	2/24	28	64.0 ± 5.7	28.1 ± 4.1	74.8	
Lin 2013	Taiwan	2007–2008	QS	5/30	35	67.7 (60, 78)	26.3 (21.2, 29.7)	24	MP
			MP	5/30	35	68.5 (55, 77)	25.9 (20, 29.5)	24	
Xu 2013	China	2009–2010	QS	7/19	35	63.5 ± 8.7	25.2 ± 3.4	24	QS
			MP	11/18	35	64.2 ± 9.3	25.2 ± 2.3	24	
Chiang 2012	Taiwan	2005	QS	3/27	38	69.7 ± 5.3	28.6 ± 3.8	24	MP
			MP	3/27	37	69.8 ± 5.4	29.6 ± 3.5	24	
Matsumoto 2011	Japan	2005–2007	QS	0/25	25	73.8 ± 1.7	Unclear	0	MP
			MP	0/25	25	73.7 ± 1.4	Unclear	0	
Yang 2010	Korea	2006–2007	QS	1/14	24	66.7 ± 6.9	Unclear	24	MP
			MP	2/14	23	68 ± 6.8	Unclear	24	
Karpman 2009	USA	2004–2005	QS	8/12	20	73 ± 7.4	28 ± 4.4	6	QS
			MP	9/10	19	73 ± 5.1	29 ± 4.6	6	
Chotanaphuti 2008	Thailand	2004–2005	QS	3/17	20	68.4 (58, 78)	Unclear	0.25	QS
			MP	4/16	20	67.5 (56, 80)	Unclear	0.25	
Shen 2007	China	2005–2006	QS	Unclear	26	Unclear	Unclear	17	QS
			MP	Unclear	33	Unclear	Unclear	17	
Huang 2007	Taiwan	2004–2005	QS	6/26	32	63 (56, 72)	Unclear	24	MP
			MP	7/28	35	65 (59, 75)	Unclear	24	
King 2007	USA	2003–2005	QS	48/52	100	67 (44, 84)	30 (22, 43)	1.5	QS
			MP	17/28	45	66 (42, 85)	32 (20, 49)	1.5	
Kim 2007	Korea	2004–2005	QS	27/93	120	65.4 (43, 88)	28.1 (19, 36)	21.5	MP
			MP	27/93	120	65.4 (43, 88)	28.1 (19, 36)	21.5	
Chin 2007	Singapore	2004	QS	6/24	30	69.0 (57, 80)	27.53 (18.6, 34.2)	Unclear	MP
			MP	3/27	30	63.4 (47, 80)	29.44 (22.7, 40)	Unclear	
Chen 2006	USA	Prior to 2002	QS	11/17	32	70 (50, 86)	28.5 (17.9, 39.9)	33	MP
			MP	11/18	38	67 (42, 81)	28.7 (21.6, 40.1)	40	
Kim 2006	Korea	2003	QS	7/65	144	68.6 (57, 85)	27.2	13.6	MP
			MP	8/64	144	67.4 (58, 84)	28.1	13.6	

*TKA* total knee arthroplasty, *BMI* body mass index, *QS* quadriceps-sparing, *MP* medial parapatellar

### Methodological quality assessment

The quality assessment of the included studies is shown in Table 2, and methodological quality was regarded as high. All eight RCTs were randomized, of which three RCTs utilized allocation concealment, four were blinded to participants and personnel, and five were blinded to outcome assessment. All the studies had incomplete data outcomes, and three selectively reported data. Observational studies achieving stars ranged from seven to eight points according to the Newcastle-Ottawa Scale, the total being nine points.

**Table 2:** Quality assessment of included studies

Study/Year	Random Sequence Generation	Allocation Concealment	Blinding of Participants and Personnel	Blinding of Outcome Assessment	Incomplete Outcome Data	Selective Reporting	Other Bias
Lin 2013^*^	Yes	Sealed envelope	Unclear	Yes	Yes	Unclear	Unclear
Xu 2013^*^	Yes	Sealed envelope	Unclear	Unclear	Yes	Unclear	Unclear
Chiang 2012^*^	Yes	Unclear	Yes	Yes	Yes	Yes	Unclear
Matsumoto 2011^*^	Yes	Unclear	Yes	Unclear	Yes	Unclear	Unclear
Yang 2010^*^	Yes	Unclear	Unclear	Yes	Yes	Yes	Unclear
Karpman 2009^*^	Yes	Unclear	Yes	Yes	Yes	Unclear	Unclear
Kim 2007^*^	Yes	Unclear	Unclear	Yes	Yes	Unclear	Unclear
Chin 2007^*^	Yes	Sealed envelope	Yes	Yes	Yes	Yes	Unclear
		Selection	Comparability		Outcomes		Total score
Huang 2016^†^		2	2		3		7
Qi 2016^†^		3	2		2		7
Chotanaphuti 2008^†^		3	2		3		8
Shen 2007^†^		3	2		3		8
Huang 2007^†^		2	2		3		7
King 2007^†^		3	2		3		8
Chen 2006^†^		3	2		2		7
Kim 2006^†^		3	2		3		8

^*^The risk of bias was assessed independently using the Cochrane Handbook for Systematic Reviews of Interventions; ^†^Methodological quality of the included studies was assessed according to Newcastle-Ottawa Scale

### Quantitative data synthesis

There were 16 studies included for meta-analysis in which there were eight RCTs [15, 16, 21, 24, 25, 32–34] and eight non-RCTs [14, 17–20, 23, 26, 35].

### Primary outcomes

The overall meta-analysis results (Table 3) were in favor of the QS approach based on long-term Knee Society (KS) function score (WMD 1.78, 95% CI 0.80 to 2.76, *P* = 0.0004, *I*^*2*^ = 0%). Furthermore, the results showed that there were no significant differences between the QS and MP approaches in the KS Knee Score beyond 24 months postoperatively (WMD -0.02, 95% CI -0.69 to 0.65, *P* = 0.95, *I*^*2*^ = 0%), in range of motion (ROM) beyond 16 months postoperatively (WMD 0.08, 95% CI -1.40 to 1.57, *P* = 0.91, *I*^*2*^ = 4%), or complications (OR 0.87, 95% CI 0.49 to 1.54, *P* = 0.63, *I*^*2*^ = 9%), infections (OR 1.53, 95% CI 0.69 to 3.39, *P* = 0.29, *I*^*2*^ = 0%), mechanical axis outliers (OR 1.05, 95% CI 0.65 to 1.72, *P* = 0.83, *I*^*2*^ = 27%), femoral component coronal angle outliers (OR 2.30, 95% CI 0.35 to 15.24 *P* = 0.39, *I*^*2*^ = 65%), tibial component coronal angle outliers (OR 0.73, 95% CI 0.40 to 1.33, *P* = 0.30, *I*^*2*^ = 40%), mechanical axis (WMD 0.35, 95% CI -0.02 to 0.73, *P* = 0.07, *I*^*2*^ = 0%), femoral component coronal angle (WMD 0.23, 95% CI -0.90 to 1.35, *P* = 0.69, *I*^*2*^ = 92%), tibial component coronal angle (WMD -0.40, 95% CI -1.29 to 0.49, *P* = 0.38, *I*^*2*^ = 92%), lateral patellar tilt (WMD -1.25, 95% CI -3.36 to 0.85, *P* = 0.24, *I*^*2*^ = 78%) or lateral patellar displacement (WMD -1.47, 95% CI -4.59 to 1.66, *P* = 0.36, *I*^*2*^ = 90%).

**Table 3:** Primary outcomes of meta-analysis results

Outcomes of Demographics	Number of Contributing Studies	Number of QS TKAs	Number of MP TKAs	WMD or OR (95% CI)	*P* - Value	Heterogeneity
KS Knee Score beyond 24 months	4	330	329	-0.02 (− 0.69, 0.65)	0.95	0%
KS Function Score beyond 24 months	3	186	185	1.78 (0.80, 2.76)	0.0004	0%
ROM beyond 16 months	6	400	404	0.08 (−1.40, 1.57)	0.91	4%
Complications	10	464	430	0.87 (0.49, 1.54)	0.63	9%
Infections	10	503	515	1.53 (0.69, 3.39)	0.29	0%
Mechanical axis outliers	5	257	266	1.05 (0.65, 1.72)	0.83	27%
Femoral component coronal angle outliers	4	237	235	2.30 (0.35, 15.24)	0.39	65%
Tibial component coronal angle outliers	5	337	280	0.73 (0.40, 1.33)	0.30	40%
Mechanical axis	5	149	147	0.35 (−0.02, 0.73)	0.07	0%
Femoral component coronal angle	6	395	395	0.23 (− 0.90, 1.35)	0.69	92%
Tibial component coronal angle	7	495	445	-0.40 (−1.29, 0.49)	0.38	92%
Lateral patellar tilt	5	418	363	-1.25 (−3.36, 0.85)	0.24	78%
Lateral patellar displacement	2	131	75	-1.47 (−4.59, 1.66)	0.36	90%

*TKA* total knee arthroplasty, *BMI* body mass index, *QS* quadriceps-sparing, *MP* medial parapatellar, *WMD* weighted mean difference, *OR* odds ratio, *CI* confidence interval, *KS* knee society, *ROM* range of motion

### Secondary outcomes

Meta-analysis showed that, when compared with the MP approach, the QS approach significantly improved ROM 1–2 weeks postoperatively (WMD 5.84, 95% CI 3.84 to 7.83, *P* < 0.00001, *I*^*2*^ = 21%), shortened length of stay (WMD -0.88, 95% CI -1.62 to − 0.15, *P* = 0.02, *I*^*2*^ = 94%) and reduced the length of incision in extension (WMD -4.62, 95% CI -6.35 to − 2.90, *P* < 0.00001, *I*^*2*^ = 99%). However, the QS approach significantly increased surgical time (WMD 12.02, 95% CI 4.06 to 19.98, *P* = 0.003, *I*^*2*^ = 95%) and tourniquet time (WMD 27.19, 95% CI 9.17 to 45.22, *P* = 0.003, *I*^*2*^ = 99%). Although the meta-analysis demonstrated significant differences in visual analogue scale (VAS) on postoperative day 1 (WMD -0.91, 95% CI -1.68 to − 0.41, *P* = 0.02, *I*^*2*^ = 81%). No other significant differences were found for secondary outcomes as shown in Table 4.

**Table 4:** Secondary outcomes of meta-analysis results

Outcomes of Demographics	Number of Contributing Studies	Number of QS TKAs	Number of MP TKAs	WMD or OR (95% CI)	*P* - Value	Heterogeneity
KS Knee Score 1.5–3 months	4	204	212	1.27 (− 0.57, 3.11)	0.18	57%
KS Function Score 1.5–3 months	4	204	212	−0.09 (−3.98, 3.81)	0.97	73%
ROM 1–2 weeks	5	148	162	5.84 (3.84, 7.83)	< 0.00001	21%
ROM 4–8 weeks	7	283	247	0.51 (−1.90, 2.91)	0.68	61%
ROM 3 months	2	152	158	−0.60 (−2.32, 1.12)	0.50	47%
ROM 12 months	2	58	68	4.00 (−5.80, 13.80)	0.42	90%
VAS 1 day	6	183	197	−0.91 (−1.68, − 0.41)	0.02	81%
VAS 3 days	2	64	70	−0.93 (−2.01, 0.14)	0.09	82%
VAS 4–8 weeks	3	84	89	−0.26 (−1.13, 0.61)	0.56	77%
Surgical time (min)	8	507	450	12.02 (4.06, 19.98)	0.003	95%
Tourniquet time (min)	8	447	462	27.19 (9.17, 45.22)	0.003	99%
Intraoperative blood loss (ml)	4	334	339	1.99 (−14.28, 18.25)	0.81	0%
Total blood loss (ml)	5	255	254	−42.94 (−150.57, 64.70)	0.43	90%
Incision, extension (cm)	7	276	284	−4.62 (−6.35, −2.90)	< 0.00001	99%
Incision, flexion (cm)	3	193	192	−1.90 (−3.99, 0.19)	0.07	99%
Length of stay (days)	8	433	441	−0.88 (−1.62, −0.15)	0.02	94%
SLR at 24 h (% of patients)	3	105	107	3.05 (0.89, 10.53)	0.08	75%

VAS, visual analogue scale; SLR, straight leg rising

### Subgroup analysis

A pooling of the RCTs is summarized in Table 5. The QS approach extended the surgical time (WMD 18.86, 95% CI 8.81 to 28.91, *P* = 0.0002, *I*^*2*^ = 94%) and tourniquet time (WMD 24.39, 95% CI 3.19 to 45.60, *P* = 0.02, *I*^*2*^ = 99%). However, the QS approach significantly improved ROM 1–2 weeks postoperatively (WMD 4.87, 95% CI 1.78 to 9.76, *P* = 0.002, *I*^*2*^ = 0%) and shortened the incision scar in extension (WMD -3.76, 95% CI -6.79 to − 0.73, *P* = 0.02, *I*^*2*^ = 99%). Furthermore, the meta-analysis of RCTs also showed that the QS approach was associated with a higher KS Function Score beyond 24 months postoperatively (WMD 1.86, 95% CI 0.86 to 2.85, *P* = 0.0002, *I*^*2*^ = 0%).

**Table 5:** Meta-analysis results of RCTs

Outcomes of Demographics	Number of Contributing Studies	Number of QS TKAs	Number of MP TKAs	WMD or OR (95% CI)	*P* - Value	Heterogeneity
KS Knee Score beyond 24 months	2	155	155	−0.18 (−1.13, 0.77)	0.71	25%
KS Function Score beyond 24 months	2	155	155	1.86 (0.86, 2.85)	0.0002	0%
ROM beyond 16 months	3	193	192	−0.41 (−2.18, 1.37)	0.65	0%
Complications	5	243	244	1.49 (0.68, 3.27)	0.32	1%
Infections	7	301	300	1.95 (0.75, 5.10)	0.17	0%
Mechanical axis outliers	2	55	54	3.80 (0.61, 23.57)	0.15	25%
Femoral component coronal angle outliers	3	93	91	5.24 (0.80, 34.28)	0.08	20%
Tibial component coronal angle outliers	3	93	91	4.14 (0.87, 19.75)	0.07	0%
Mechanical axis	3	88	89	0.34 (−0.37, 1.05)	0.35	15%
Femoral component coronal angle	5	251	251	0.07 (−1.30, 1.44)	0.92	93%
Tibial component coronal angle	5	251	251	−0.31 (− 1.58, 0.97)	0.64	93%
Lateral patellar tilt	2	143	144	0.73 (−0.30, 1.76)	0.16	0%
KS Knee Score 1.5–3 months	3	178	179	1.01 (−0.74, 2.76)	0.26	61%
KS Function Score 1.5–3 months	3	178	179	−0.67 (−5.45, 4.10)	0.78	82%
ROM 1–2 weeks	2	58	56	4.87 (1.78, 7.96)	0.002	0%
ROM 4–8 weeks	3	93	91	1.68 (−2.16, 5.51)	0.39	60%
VAS 1 day	3	93	91	−0.07 (−0.49, 0.35)	0.74	0%
VAS 4–8 weeks	2	58	56	−0.46 (−2.31, 1.40)	0.63	87%
Surgical time (min)	5	243	241	18.86 (8.81, 28.91)	0.0002	94%
Tourniquet time (min)	3	193	192	24.39 (3.19, 45.60)	0.02	99%
Intraoperative blood loss (ml)	2	158	157	3.10 (−24.89, 31.09)	0.83	0%
Total blood loss (ml)	4	111	110	4.24 (−56.29, 64.77)	0.89	48%
Incision, extension (cm)	4	188	188	−3.76 (−6.79, −0.73)	0.02	99%
Incision, flexion (cm)	3	193	192	−1.90 (−3.99, 0.19)	0.07	99%
Length of stay (days)	4	205	204	−0.34 (−1.02, 0.34)	0.33	71%
SLR at 24 h (% of patients)	2	73	72	1.62 (0.79, 3.30)	0.19	0%

*TKA* total knee arthroplasty, *QS* quadriceps-sparing, *MP* medial parapatellar, *WMD* weighted mean difference, *OR* odds ratio, *CI* confidence interval, *KS* knee society, *ROM* range of motion, *VAS* visual analogue scale, *SLR* straight leg rising

### Publication bias

**Fig. 2:**
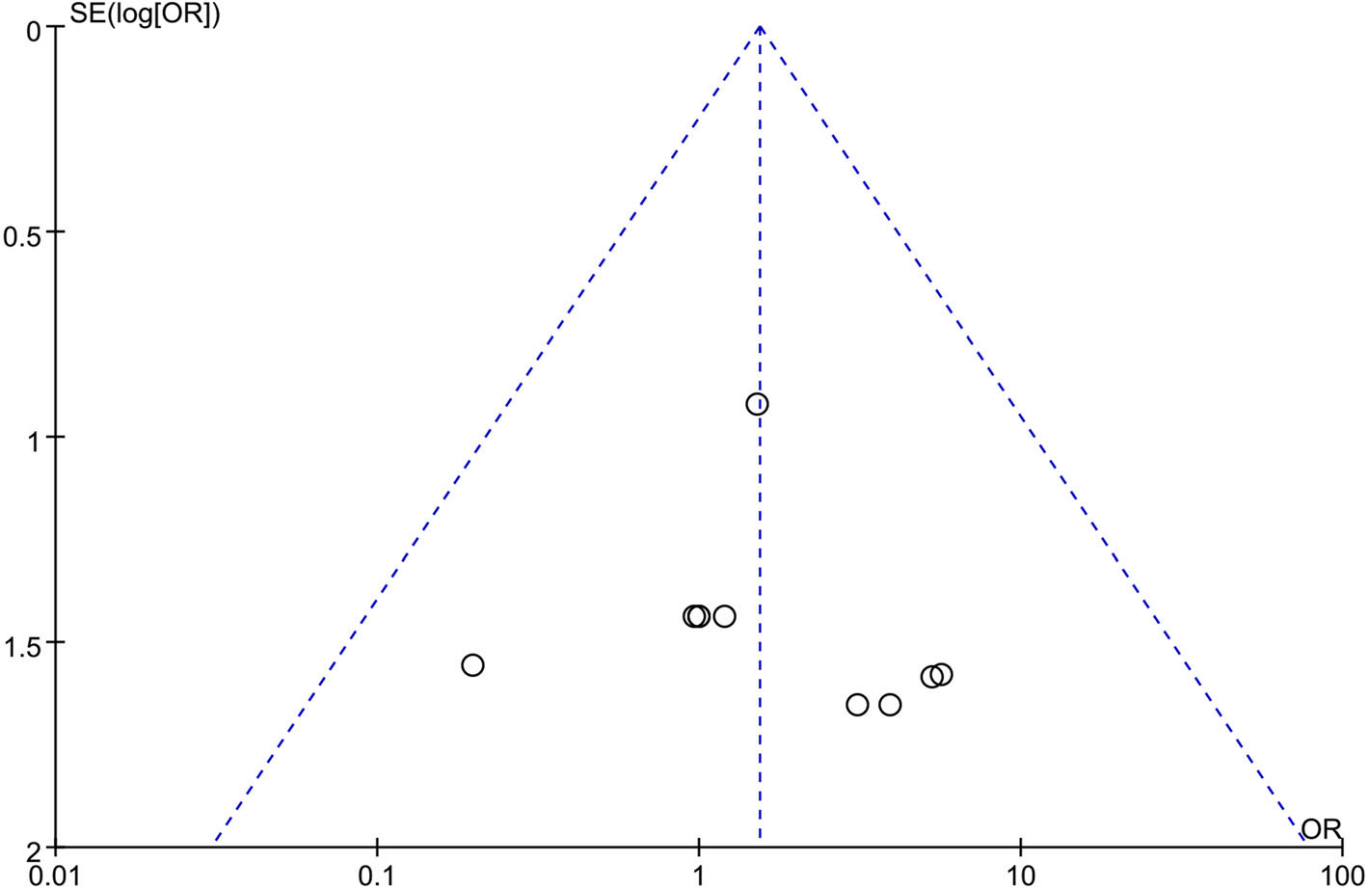
Funnel plot illustrating meta-analysis of infections

Figure 2 shows a funnel plot of the studies included in this meta-analysis that reported infections. All studies lie inside the 95% CIs, with an even distribution around the vertical, indicating no obvious publication bias.

## Discussion

The results suggest that QS approach may be associated with higher KS function score beyond 24 months postoperatively, could improve ROM 1–2 weeks postoperatively, and shorten incision (extension) with significantly longer surgical and tourniquet time in both overall and subgroup meta-analysis.

According to both the subgroup meta-analysis of RCTs and the overall meta-analysis, results showed that the QS approach was favored in terms of the KS function score beyond 24 months postoperatively which was a primary outcome with a WMD 1.78 and 1.86, respectively. However, we cautiously thought that QS approach could not be confirmed as superior because Lee et al. [36] found that a minimal clinically important difference (MCID) in the KS function score was between 6.1 and 6.4. Besides, QS approach significantly improved ROM 1–2 week postoperatively and shortened the incision length in extension. Longer surgical time and tourniquet time were needed in QS group without increasing complications and infections. Based on these results of the secondary outcomes, we identified that the QS approach may accelerate early recovery to some extent and improved cosmesis which may make patients to be more satisfied with their surgery without increasing the risk of surgery. But it is undeniable that a longer surgery time may lead to increased hospital costs.

In our overall meta-analysis, the QS approach had significant advantages over the MP approach on VAS 1 day postoperatively and length of stay, which were not identified in the subgroup meta-analysis of RCTs. Although the WMD was statistically significant, it falls below the threshold for clinical significance according to the MCID of VAS [37]. Therefore, the possibility that QS approach may accelerate early recovery was supported to a limited extent.

Meanwhile, we observed that the QS approach was not associated with a higher risk of malalignment of low limb and poor position of prosthesis, which was demonstrated in both overall and subgroup meta-analysis. The importance of accurate lower limb alignment and prothesis position after TKA and the greater risk of implant failure with malalignment have been well recognized [38, 39]. Owing to the importance of those factors, we should pay attention to this situation even though meta-analysis did not identify this issue. As arthroplasty surgeons know, the QS approach can easily be extended or converted to the MP approach during the surgery. Therefore, if a surgeon is not sufficiently skilled in the TKA procedure, the QS approach should be appropriately extended to ensure good bone resection and prosthesis installation.

The findings from our meta-analysis are in partial disagreement with the results and conclusions of two recent meta-analyses by Peng et al. [40] and Kazarian et al. [41]. The disagreements are not only due to differences in the concluded articles and the extraction and selection of data; they are also due to differences in the included articles of RCTs. In our view, the meta-analysis by Peng et al. included three studies that did not meet the inclusion criteria and excluded two articles that met the inclusion criteria. In the included studies of Peng et al., Shen et al. [18] was a cohort study, Tasker et al. [42] compared the mini-midvastus or subvastus approach to the MP approach and Lin et al. [43] compared the QS approach to the mini-MP approach. In addition, Peng et al. did not include two studies [15, 21] that met the inclusion criteria of the meta-analysis. For the meta-analysis by Kazarian et al., we considered that an article by Yang et al. [15] met the inclusion criteria even though it was not included and a study comparing the QS approach with the mini-MP approach by Lin et al. [43] was enrolled. Because these deviations could potentially affect some of the results, they might provide an explanation for the partial disagreement between our meta-analyses.

The inclusion of both RCTs and retrospective comparative studies enhanced the sample size and robustness of the estimates when compared with previous studies [40, 41]. Although a meta-analysis of RCTs only would be ideal, the limited number of RCTs and their size limits the scope of this review and prevents its findings from being conclusive.

Between-study heterogeneity was found to exist with some outcomes. Included studies adopted different research objects, research designs, and measurement of results, differences, all of which may contribute to the significant between-study heterogeneity. After careful analysis of these documents, we found that design and objects were also potential contributors to heterogeneity. Therefore, we conducted a subgroup meta-analysis pooling only RCTs to increase the reference value of the results. It is well known that RCTs standardize the research process through randomization, blinding, strict quality control, etc. to obtain reliable research results. In addition, if heterogeneity persisted, we adopted random-effects model to potentially reduce, but not abolish, the effect of heterogeneity. The limitation is that the duration of follow-up of these studies is still not long enough. A follow-up period of more than ten years is required to evaluate and confirm outcomes, especially regarding relationships between the mechanical axis, prosthesis position and functional scores.

## Conclusions

In summary, the use of QS approach in patients undergoing TKAs appears to be effective in improving ROM 1–2 postoperatively and reducing the length of incision in knee extension. In addition, the overall meta-analysis illustrated that QS approach may shorten the length of stay. However, the QS approach also significantly increases surgical and tourniquet time. Apart from this, the two surgical techniques appear to be equivalent in other aspects such as mechanical axis, prosthesis position, complications, infections and so on. On the basis of these findings, we are optimistic about the QS approach to some extent.

## Abbreviations

BMI: body mass index
CI: Confidence interval
DVT: Deep vein thrombosis
KS: Knee society
MIS-TKA: Minimally invasive surgery-total knee arthroplasty
MP TKA: Medial parapatellar total knee arthroplasty
NOS: Newcastle-Ottawa Scale
OR: Odds ratio
PRISMA: Preferred Reporting Items for Systematic Reviews and Meta-analysis
QS TKA: Quadriceps-sparing total knee arthroplasty
RCT: Randomized controlled trial
ROM: Range of motion
SLR: Straight leg raising
TKA: Total knee arthroplasty
VAS: Visual analog scale
WMD: Weighted mean difference
